# Prolonged light exposure time enhances the photosynthetic investment in osmotrophic *Ochromonas*

**DOI:** 10.1128/aem.01032-25

**Published:** 2025-06-24

**Authors:** Xiaoqing Xu, Xiaoyu Cheng, Zhihao Shao, Zhou Yang, Lu Zhang

**Affiliations:** 1 Jiangsu Key Laboratory for Biodiversity and Biotechnology, School of Biological Sciences, Nanjing Normal University 12534, Nanjing, China; Georgia Institute of Technology, Atlanta, Georgia, USA

**Keywords:** photoperiod, osmotrophy, carbon acquisition, metabolic plasticity, photosynthesis

## Abstract

The photoperiod, as a critical external environmental signal, triggers a cascade of signaling responses in organisms that significantly affect photosynthetic efficiency and photomorphogenesis in autotrophs, while also influencing behavioral patterns and activity rhythms of heterotrophs. Despite its importance, the mechanisms by which mixotrophs respond to photoperiod changes remain largely unexplored. It is crucial for understanding metabolic plasticity how mixotrophs respond to light availability and make optimal decisions during diurnal transitions by regulating their autotrophic and osmotrophic pathways. Therefore, this study focused on *Ochromonas gloeopara*, a eukaryotic protist capable of both photoautotrophic and osmotrophic growth, aiming to explore the metabolic strategies of mixotrophs in response to changes in photoperiod. The results showed the following. (i) Under autotrophic conditions, the optimal photoperiod for photosynthetic efficiency in *Ochromonas* was approximately 12 h of light exposure, while prolonged light exposure beyond this duration reduced photosynthetic investment and efficiency, accompanied by an increase in heat dissipation to prevent photodamage. (ii) Under osmotrophic conditions, *O. gloeopara* adapted to prolonged light exposure by reducing. The reliance on external organic carbon sources and enhancing photosynthetic capacity, thereby shifting towards a more autotrophic metabolic mode. This study systematically elucidates the nutritional strategies of mixotrophic *O. gloeopara* in response to photoperiod changes at the levels of population dynamics, photosynthetic physiology, and carbon acquisition pathways, deepening our understanding of the response to photoperiodic changes in mixotrophs. These findings provide important theoretical insights for understanding the functional roles of mixotrophs in ecosystems and for accurately predicting changes in global carbon cycles.

**IMPORTANCE:**

Mixotrophs possess flexible metabolism modes and multiple ecological roles, making them sensitive to environmental changes. Due to their widespread distribution and unique nutritional strategy, they serve as key functional groups in marine and freshwater ecosystems, with significant roles in global biogeochemical cycles. Photoperiod, a critical environmental cue, regulates circadian rhythms and may influence the metabolic strategies of mixotrophs. Therefore, this study focused on how the mixotrophic microorganisms *Ochromonas gloeopara* adjusted autotrophic and osmotrophic pathways in response to photoperiodic changes. These findings highlight the metabolic flexibility of mixotrophic organisms in response to photoperiodic changes, providing new insight on how mixotrophs regulate the flow of materials and reshape the food web structures. This research offers valuable and innovative perspectives for understanding the functional roles of mixotrophic microorganisms in ecosystems, with important implications for improving the accuracy of global carbon cycle predictions.

## INTRODUCTION

Within biological organisms, there exists an intrinsic circadian regulatory system that orchestrates physiological, molecular, and neural signals. This system enables the organism to adapt to environmental changes, ensuring that physiological metabolism and behavioral activities remain in harmony with the external environment (1–3). In natural environments, the photoperiod—referring to the duration of daily light exposure—exhibits substantial variability with latitude and season. For instance, at high latitudes, it can shift from complete darkness during the winter solstice to continuous light throughout the summer (4). Furthermore, there is a close relationship between the length of the photoperiod and temperature. During winter, the shorter photoperiod is typically accompanied by lower temperatures, leading organisms to enter a state of dormancy or reduce their metabolic rates. In contrast, the longer photoperiod and higher temperatures in summer promote growth and reproductive activities in both plants and animals (5, 6). Thus, the photoperiod is considered a crucial external environmental signal for organisms to influence their intrinsic circadian rhythms, mainly through photoreceptors and light-sensitive receptors detecting photoperiod changes and triggering a series of signal transduction cascades that significantly impact growth, development, and physiological metabolism (7, 8).

In aquatic ecosystems, changes in the photoperiod affect the life stages of nearly all the organisms. For instance, photoperiod affects the ability of phototrophic organisms to capture light energy and carbon fixation (9–11), thereby interfering with light-driven biological processes including cell division, nutrient uptake, growth, and the repair of damaged photosystem II, and these processes are highly related to their circadian rhythms (12, 13). Phytoplankton exhibits optimal photosynthetic efficiency under specific light durations, and varying photoperiods directly influence their photosynthesis and carbon storage processes (14). Shorter photoperiods reduce the total light exposure during the day, which in turn leads to a decreased growth rate of aquatic primary producers (15). Under longer photoperiods, non-photochemical quenching (NPQ) of the excited chlorophyll state is crucial for protecting the photosynthetic apparatus from light-induced damage, helping to maintain the stability of photosynthesis. For heterotrophic organisms, the effects of photoperiod predominantly influence their growth, grazing, reproduction, and behavioral patterns (16–18). Jakobsen and Strom (19) found that growth and grazing rates of four types of heterotrophic marine protists exhibit distinct diel cycles. Additionally, high temperature and longer light exposure increased total offspring and heart rate in *Moina micrura* (20). Therefore, photoperiod has profound effects on the biomass and the transfer of primary production in aquatic ecosystems.

Mixotrophs are a common group of protists characterized by their extraordinary ability to utilize multiple sources of carbon and energy, supported by highly flexible metabolic strategies, typically including phototrophy (light-driven autotrophy) and heterotrophy via either phagotrophy (particle ingestion) or osmotrophy (uptake of dissolved organic compounds) (21–24). While phagotrophy is widely recognized as the defining trait that distinguishes planktonic mixotrophs from strictly autotrophic phytoplankton (25), osmotrophy remains a metabolically significant and ecologically successful strategy in many mixotrophic protists (26). Therefore, investigating osmotrophic capacity and regulation is essential for understanding the metabolic plasticity in mixotrophs.

This flexibility supports their broad geographical distribution, spanning a wide range of ecosystems from freshwater to marine environments and latitudinal gradients from the tropics to the poles (27, 28). Under changing environmental conditions, it can shift the trade-offs between autotrophy and heterotrophy through adjusting phototrophic, phagotrophic, and osmotrophic pathways. For instance, warming can lead to an increased reliance on heterotrophy in mixotrophic organisms (29), while enhanced inorganic carbon enhances the autotrophic capability of mixotrophic *Euglena gracilis* (30). Light availability and intensity are key factors shaping mixotrophic organisms’ metabolic modes. For instance, light exposure enhanced photosynthesis, phagotrophy, and tetrapyrrole synthesis, and prolonged exposure to darkness increased feeding capacity, which was correlated with decreases in photosynthetic capacity (31). Thus, mixotrophic organisms perform strong plasticity in responding to changes in light conditions. Nevertheless, photoperiod, as one of the important light conditions, not only affects the photoreceptive processes of photoautotrophic organisms but also influences the heterotrophic organisms. Therefore, mixotrophic organisms, exhibiting both biological processes, will display response characteristics different from those of organisms with a single nutritional strategy. This is crucial for understanding the metabolic plasticity of mixotrophs under changing light conditions.

Although photoperiod variations in natural environments are typically accompanied by temperature fluctuations, numerous studies have investigated the impact of light availability on mixotrophic organisms, primarily focusing on the binary comparison between light and dark conditions. However, light duration, as a critical variable of light availability, provides a more comprehensive understanding of how mixotrophic organisms dynamically adapt to changes in light exposure. Accordingly, this study aims to systematically examine the influence of light duration on *O. gloeopara* under different nutritional modes, within controlled temperature conditions, to elucidate their adaptive responses to photoperiod fluctuations.

Herein, a common representative mixotrophic organism *O. gloeopara* was selected as our tested organism, and thus the photosynthetic physiology, morphology, and population characteristics of *O. gloeopara* under different photoperiods and nutritional modes were investigated as the indices of their nutritional strategies. We then formulated the following hypotheses: (i) Autotrophic *O. gloeopara* has an optimal light exposure duration for photosynthetic efficiency, and as light exposure time increases, these organisms reduce their photosynthetic investment and efficiency while enhancing their thermal dissipation to prevent light damage, and (ii) Osmotrophic *O. gloeopara* adapts to extended light exposure time by reducing the uptake of external organic carbon sources and enhancing chlorophyll synthesis, thereby increasing the relative contribution of photosynthesis.

## RESULTS

### Population dynamics

**Fig 1:**
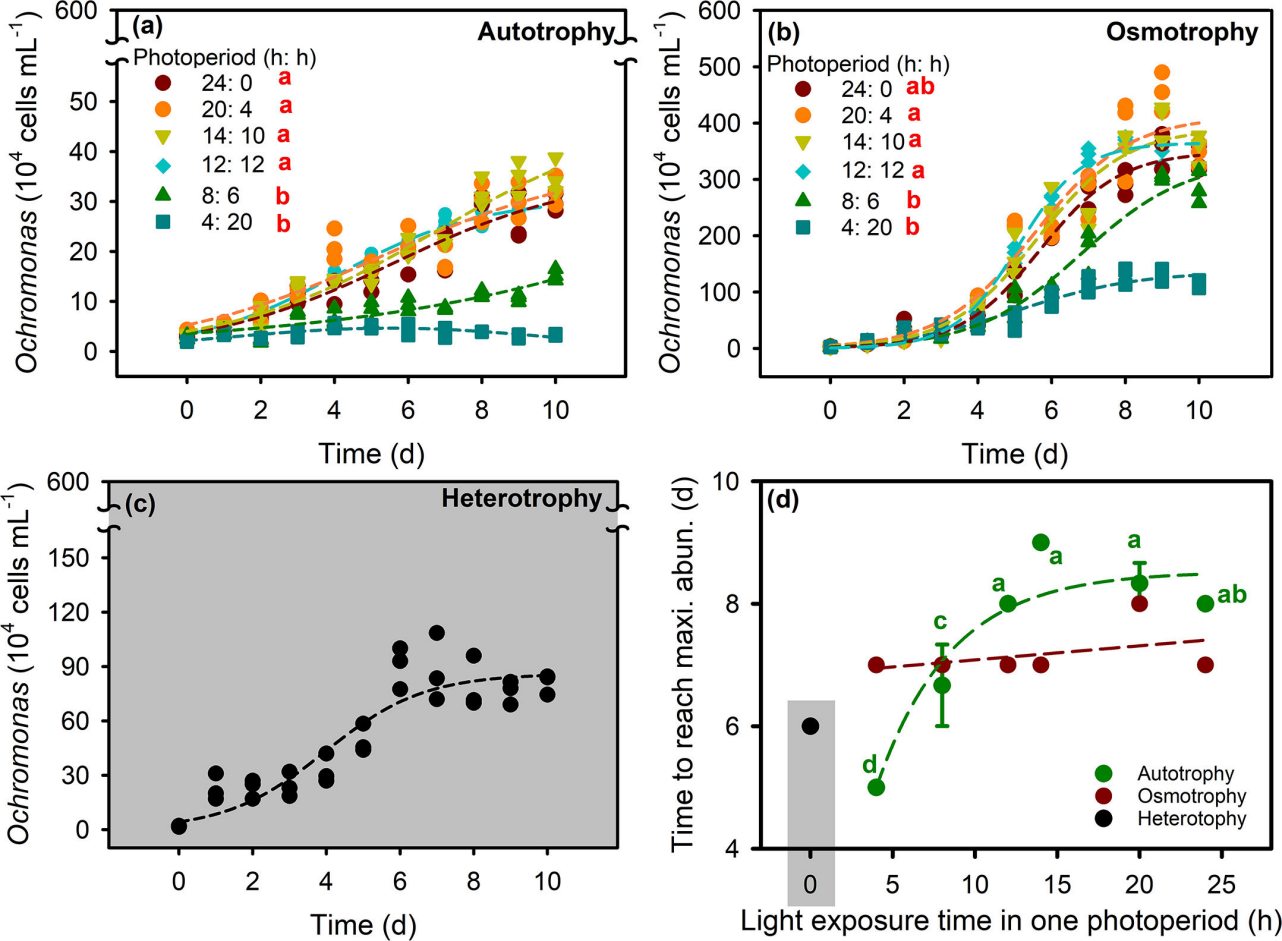
Population dynamics of *O. gloeopara* of different nutritional modes under different photoperiods. (**a**) Autotrophic *O. gloeopara*, (**b**) osmotrophic *O. gloeopara*, (**c**) heterotrophic *O. gloeopara*, and (**d**) the time for *O. gloeopara* to reach the maximum density.

**Fig 2:**
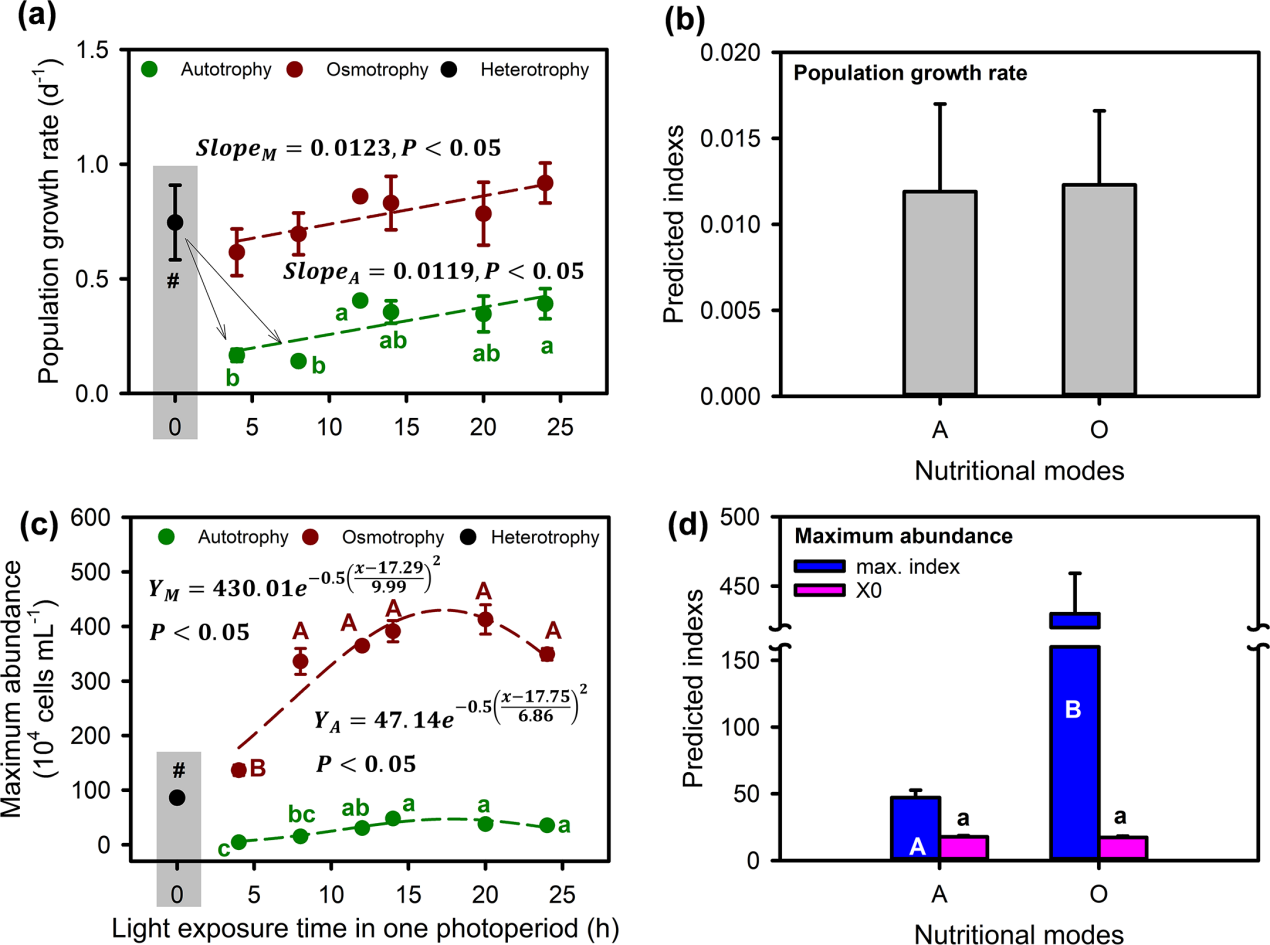
Growth performances of *O. gloeopara* cultured at different metabolic modes under different photoperiods. (**a**) Population growth rates, (**b**) predicted slope of population growth rate with light exposure time, (**c**) dynamics of maximum population abundance with light exposure time, and (**d**) maximum values of maximum population of autotrophic and osmotrophic *O. gloeopara* and its corresponding light exposure time.

Photoperiod had a significant effect on the population increase of *O. gloeopara* under autotrophic and osmotrophic growth conditions (Fig. 1a and b, *P* < 0.05). The autotrophic *O. gloeopara* was significantly lower under the 8 h:16 h and 4 h:20 h photoperiods compared with the other photoperiods. There was no significant difference in the time that populations reached the maximum value of osmotrophic *O. gloeopara* under various photoperiod conditions, while it increased with increasing photoperiod for autotrophic *O. gloeopara* (Fig. 1d). Both population growth rates of *O. gloeopara* grown under autotrophic and osmotrophic conditions increased linearly with the photoperiods increased, and increase rates were not significantly different between these two nutritional modes (Fig. 2a and b). When the light duration was about 17 h, the maximum density of *O. gloeopara* under autotrophic and osmotrophic nutritional modes reached the maximum values of 4.8 × 10^5^ and 4.3 × 10^6^ cells mL^−1^, respectively (Fig. 2c and d).

### Cell volume and morphology

**Fig 3:**
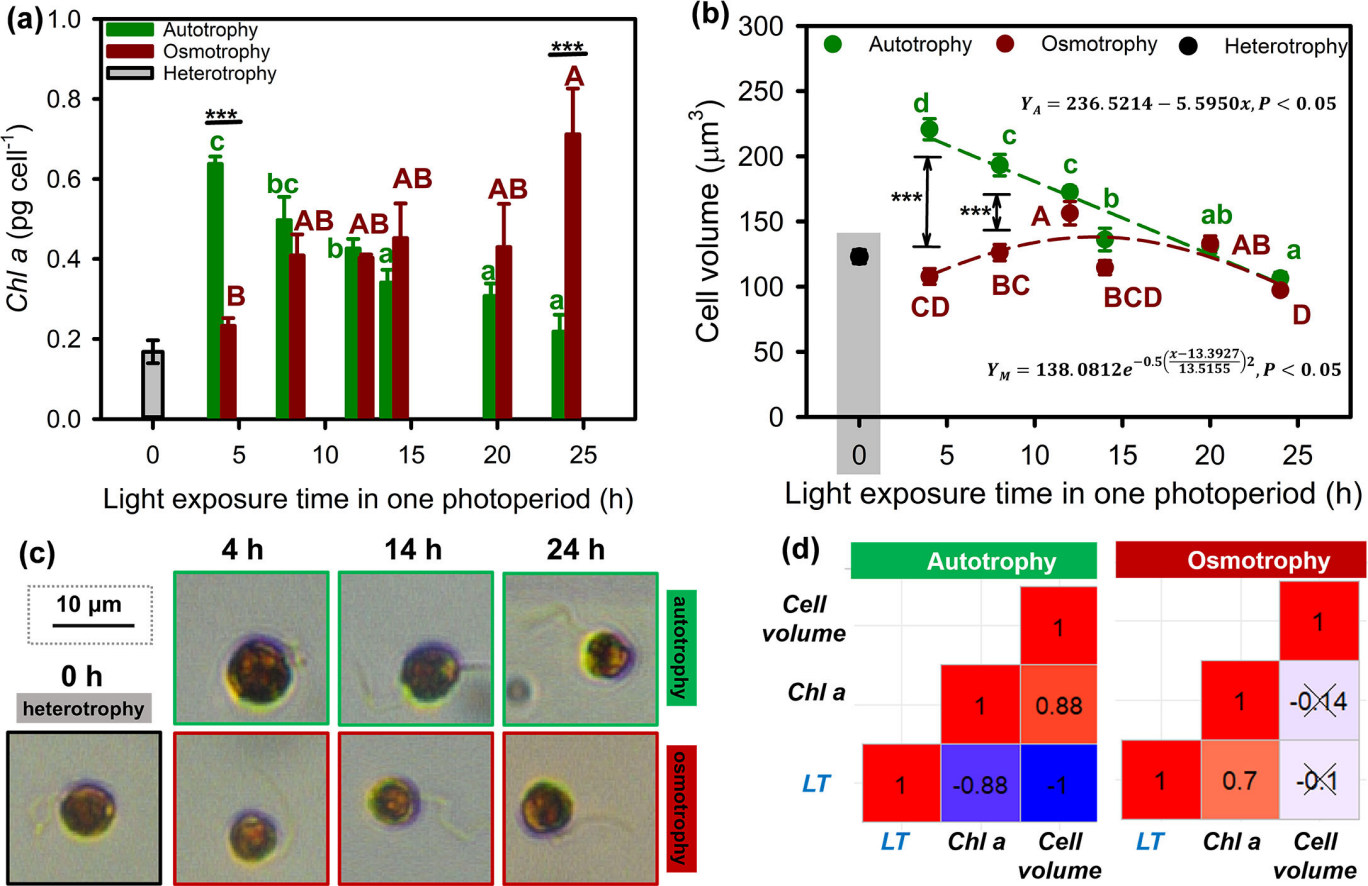
Morphology-related characteristics of *O. gloeopara* under different metabolic modes and photoperiods. (**a**) Chlorophyll content per cell, (**b**) cell volume, (**c**) micrographs of cell morphology, and (**d**) correlation analysis among photoperiods, chlorophyll content per cell, and cell volume.

The results of two-way analysis of variance (ANOVA) showed that the chlorophyll *a* content per cell and the cell volume of *O. gloeopara* were significantly affected by photoperiods and nutritional modes and their interaction effect (Table S1, P < 0.05). The chlorophyll *a* content and cell volume of autotrophically grown *O. gloeopara* decreased with the increase of daylight exposure time in the photoperiod. In contrast, *O. gloeopara* under osmotrophic conditions gradually increases their chlorophyll *a* content per cell with the increasing light exposure time, and their cell volume showed a trend of first increasing and then decreasing, reaching a maximum value at a light exposure time of approximately 13.4 h (Fig. 3a through c). At light exposure times of 5 and 10 h, autotrophic *O. gloeopara* significantly had larger size than that under osmotrophic status (Fig. 3b, *P* < 0.001). Correlation analysis indicated a significant negative correlation between photoperiod and both the chlorophyll *a* content and cell volume of autotrophically grown *O. gloeopara* (*P* < 0.05), as well as a significant correlation between cell volume and chlorophyll *a* content (*P* < 0.05). Moreover, there was only a positive correlation between light exposure time and chlorophyll *a* content for osmotrophic *O. gloeopara* (*P* < 0.05).

### Photosynthetic performances

**Fig 4:**
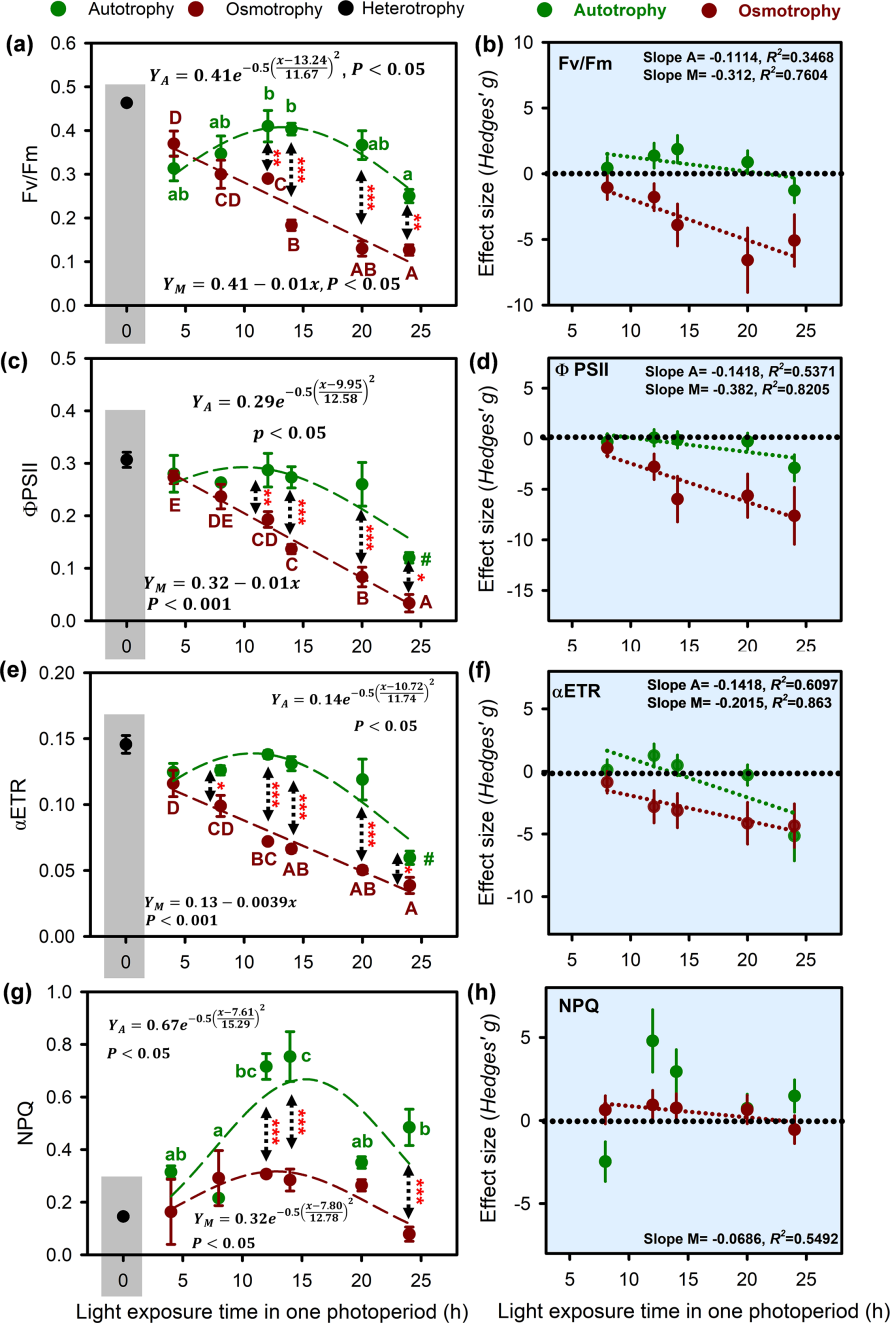
Photosynthetic efficiency of *O. gloeopara* under different metabolic modes (a, c, e, and g) and the effect size of photoperiod on these indicators (b, d, f, and h).

*O. gloeopara* grown under autotrophic conditions exhibited an optimal photoperiod (*O. gloeopara* showed best physiological or metabolic state) response characteristic of photosynthetic efficiency (Fig. 4), with predicted maximum values for Fv/Fm, ΦPSII, and αETR of 0.41, 0.29, and 0.14, respectively, at light exposure time of approximately 11.6, 12.5, and 11.74 h. However, under osmotrophic growth conditions, the photosynthetic efficiency of *O. gloeopara* decreased as the daylight time of photoperiod increased. In particular, the photosynthetic efficiency of osmotrophic *O. gloeopara* was significantly higher when light exposure time was less than 14 h, compared with that of 20 and 24 h (*P* < 0.05). Moreover, when the light exposure time exceeded 14 h, the photosynthetic efficiency of autotrophic *O. gloeopara* was significantly higher than that of osmotrophic *O. gloeopara* (*P* < 0.05). Furthermore, the NPQ of autotrophic and osmotrophic *O. gloeopara* exhibited a trend of initial increase followed by a decrease with light exposure time and reached their predicted maximum values at approximately light exposure time of 15.29 and 12.78 h (Fig. 4g). Additionally, there was a significant difference in the NPQ of *O. gloeopara* under both autotrophic and heterotrophic conditions when the photoperiod was 12, 14, and 24 h (*P* < 0.05). *O. gloeopara* under heterotrophic conditions still retained the capacity for photosynthesis, with a photosynthetic efficiency significantly higher than that of osmotrophic growth conditions.

**Fig 5:**
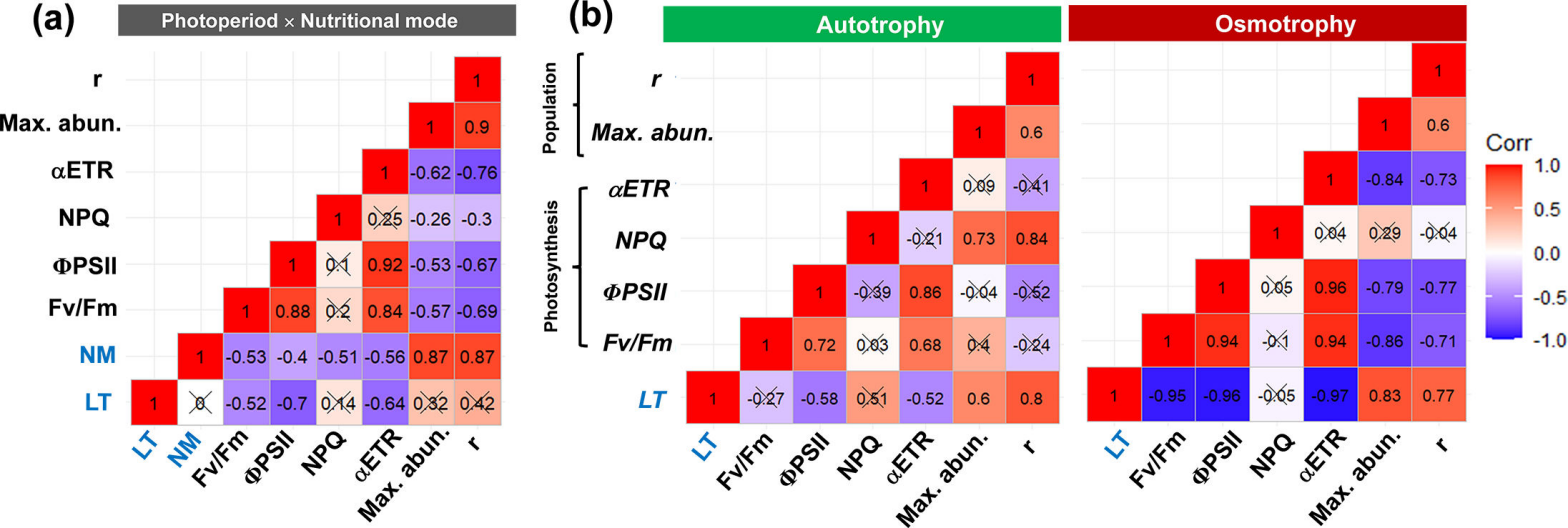
Correlation among photoperiod (LT), nutritional modes (NM), photosynthetic efficiency, and population indicators of *O. gloeopara*.

The results of the two-way ANOVA suggested that both the nutritional mode and photoperiod had significant separate and interactive impacts on the photosynthetic efficiency of *O. gloeopara* (Table S1, *P* < 0.01). To further understand the impact of photoperiod changes on the photosynthetic efficiency of *O. gloeopara* under different metabolic modes, the effect size analysis indicated that the impact of photoperiod on the NPQ of osmotrophic *O. gloeopara* gradually approached zero as the light exposure time increased (Fig. 4h). In contrast, the impact of photoperiod on the Fv/Fm, ΦPSII, and αETR gradually increased, indicating a stronger inhibitory effect (Fig. 4b, d and f). The effect size of the increase in light exposure time on the photosynthetic efficiency of autotrophic *O. gloeopara* was approximately zero, indicating no significant impact. Correlation analysis demonstrated that compared with that of autotrophic *O. gloeopara*, the photosynthetic efficiency of osmotrophic *O. gloeopara* exhibited a stronger correlation with photoperiod, indicating that osmotrophic *O. gloeopara* was more susceptible to changes in photoperiod (Fig. 5).

### Carbon utilization capacity

**Fig 6:**
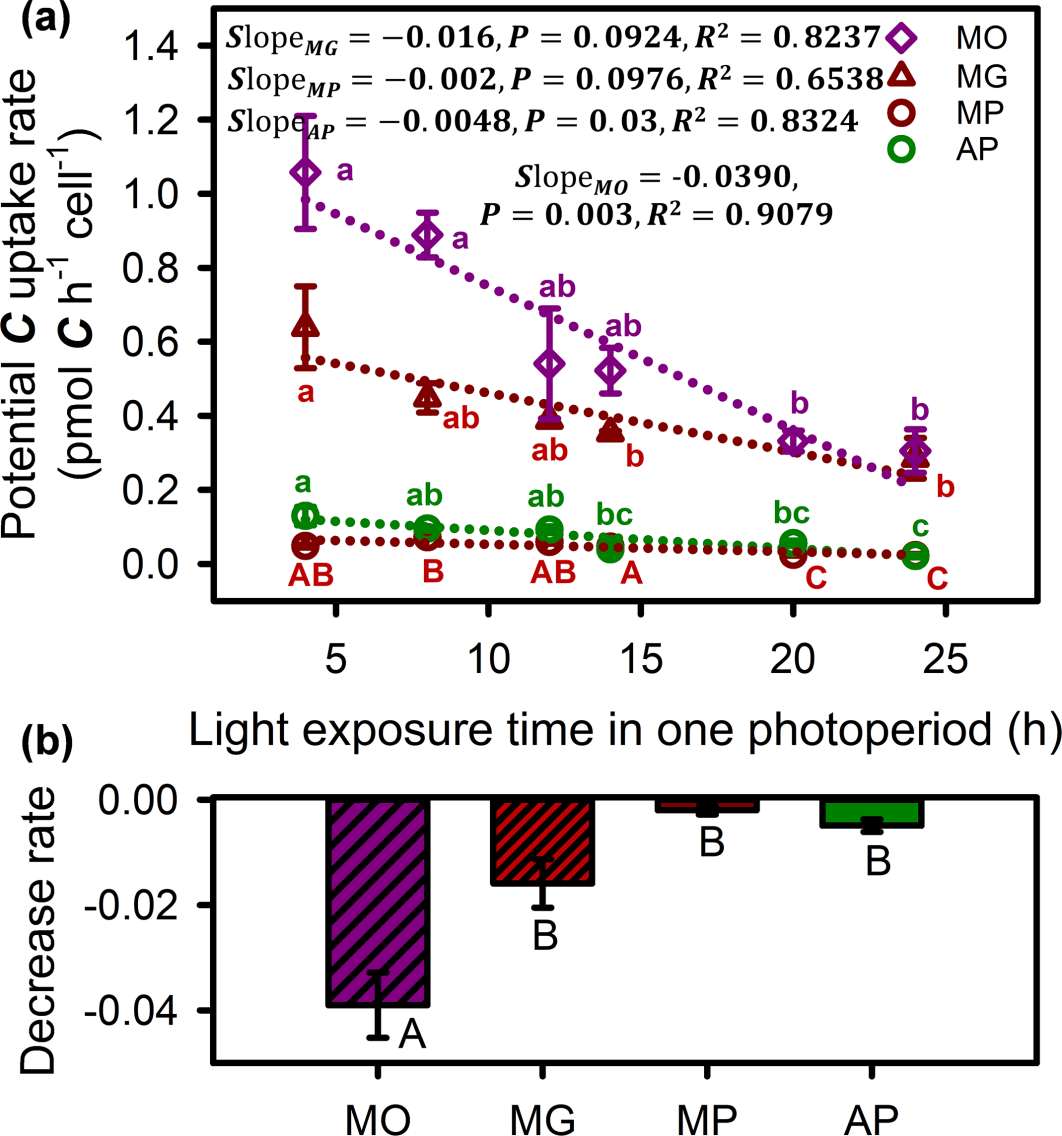
Carbon utilization strategies of *O. gloeopara* under different photoperiod (time unit: h). Carbon acquisition rates of autotrophic and osmotrophic *O. gloeopara* under different light exposure time (**a**) and the change rate in carbon acquisition rates with light exposure time (**b**). AP, potential photosynthetic carbon fixation rate under autotrophic conditions; MP, potential photosynthetic carbon fixation rate under osmotrophic conditions of mixotrophs; MG, carbon acquired through phagotrophy of mixotrophs; MO, carbon acquired through osmotrophy of mixotrophs.

Nutritional mode and photoperiod had significant interaction effects on potential carbon fixation of *O. gloeopara* (Table S1, *P* < 0.05). The potential photosynthetic carbon fixation rate of autotrophic and osmotrophic *O. gloeopara* shows a significant linear decline with the increase in light exposure time (Fig. 6a). The carbon acquisition rate through phagotrophy decreased with increasing light exposure time, consistent with the trend observed in osmotrophic *O. gloeopara*. This further demonstrates that prolonged light exposure inhibits the uptake of organic carbon in *O. gloeopara*. Moreover, the change rate in carbon acquisition by osmotrophy was significantly higher than that by photosynthetic carbon fixation for osmotrophic *O. gloeopara*, suggesting that fluctuations in the photoperiod had a relatively minor impact on the carbon fixation capability of *O. gloeopara* through photosynthesis (Fig. 6b).

**Fig 7:**
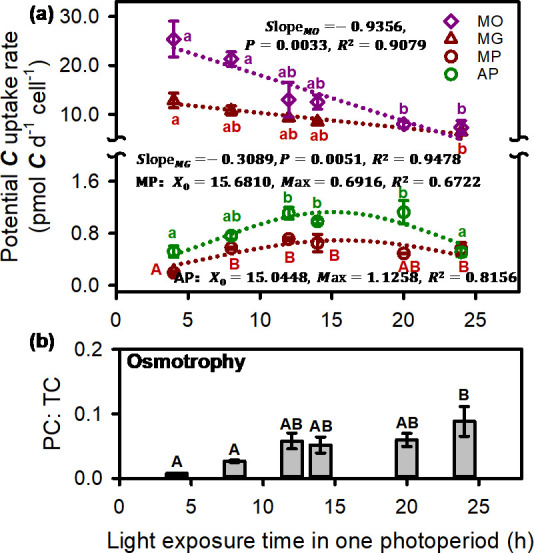
Carbon utilization strategies of *O. gloeopara* under different photoperiod (time unit: days). Carbon acquisition rates of autotrophic and osmotrophic *O. gloeopara* under different light exposure time (**a**) and the ratio of potential photosynthetic carbon fixation to total carbon acquisition (**b**). PC, potential carbon fixation rates by photosynthesis; TC, total of potential photosynthetic carbon fixation and carbon acquired through osmotrophy.

Furthermore, the carbon acquisition rates were further converted to a daily basis. The results showed that the potential photosynthetic carbon fixation rates of autotrophic and osmotrophic *O. gloeopara* increased firstly and then decreased (Fig. 7a), and their carbon fixation rates reached the maximum at light exposure times of 15 and 16 h, with values of 1.1 and 0.6 pmol C d⁻¹ cell⁻¹, respectively. Except for the continuous light conditions, the photosynthetic carbon fixation rate of autotrophic *O. gloeopara* was significantly higher than that under osmotrophic condition (Fig. 7a, *P* < 0.05). Additionally, in osmotrophic *O. gloeopara*, the proportion of potential photosynthetic carbon fixation to total carbon acquisition increased with light exposure times (Fig. 7b).

## DISCUSSION

Light and food availability are critical drivers influencing the metabolic mode shifts in mixotrophs (32, 33). Light not only provides the energy required for photosynthesis but also acts as a key environmental signal regulating organismal physiology and development (34). Moreover, light plays an essential role in regulating photomorphogenesis in plants (35), and variations in light duration influence the time for plants to receive light energy, thereby regulating both photosynthesis and circadian rhythms in autotrophic organisms (36). In this study, the growth of *O. gloeopara* relied on light availability under both autotrophic and osmotrophic conditions. However, the responses of *O. gloeopara* to photoperiod exhibited significant differences between these two metabolic modes. The following sections provide a detailed analysis from the perspectives of photosynthetic physiology, carbon acquisition pathways, and regulatory strategies at the individual and population levels.

### Growth characteristics of *O. gloeopara* in response to increased light exposure time of photoperiod

As the duration of light exposure increased, the cell volume of *O. gloeopara* under autotrophic growth gradually decreased, accompanied by reductions in chlorophyll content and photosynthetic efficiency. The smaller cell size corresponds to lower metabolic demands (37). Thus, *O. gloeopara* exposed to prolonged light conditions may achieve population growth by reducing cell volume, thereby lowering the high metabolic investment required under autotrophic conditions. This phenomenon has also been observed in the study of *Ankistrodesmus falcatus*, where cells exhibited smaller size under prolonged light exposure and larger size under dark conditions (38). In addition to influencing cell morphology and metabolic investment, variations in light exposure time may regulate the metabolic modes of *O. gloeopara* through circadian rhythms. In plant cells, light-sensitive receptors, such as phytochromes and cryptochromes, activate circadian systems, regulating downstream gene expression and maintaining metabolic homeostasis (39). Similar light-regulated mechanisms may exist in mixotrophs, coordinating resource allocation between autotrophic and osmotrophic pathways (40). Moreover, with the addition of dissolved organic carbon, osmotrophic *O. gloeopara* under long light exposure showed no significant difference in cell size compared with autotrophic *O. gloeopara*; however, chlorophyll content was higher and aligned with the enhanced carbon fixation rate and decreased dissolved organic carbon uptake rate. These findings suggest that osmotrophic *O. gloeopara* under long photoperiods preferentially allocated resources toward autotrophic metabolism while reducing reliance on osmotrophy. Furthermore, our results showed the growth rate and maximum population size of osmotrophic *O. gloeopara* under long photoperiods were higher than those under short photoperiods. Thus, for osmotrophic *O. gloeopara*, prolonged light exposure time increased the allocation of resources to autotrophic metabolism, implying enhanced energy contribution of autotrophy to the growth of mixotrophs. In contrast, under short photoperiods, although osmotrophic utilization of organic carbon increased, the energetic burden of maintaining photosynthetic apparatus under limited light likely offset the benefits (41). For plant cells, mitochondrial respiration usually tends to metabolize the direct products (e.g., monosaccharides) from photosynthesis, as catabolizing ingested organic carbon for respiration is a relatively high-cost pathway (42). Therefore, osmotrophic *O. gloeopara* acquired energy by reducing osmotrophy and increasing the allocation to autotrophy, which may serve as a better survival strategy under long light periods.

### Regulation strategy in photosynthesis in *O. gloeopara* under photoperiod variation

The photosynthetic efficiency of osmotrophic *O. gloeopara* significantly decreased with increasing light duration, whereas autotrophic *O. gloeopara* exhibited optimal photoperiod for photosynthetic efficiency was approximately 12 h of light exposure. Meanwhile, there were distinct investment strategies in the photosynthetic apparatus between osmotrophy and autotrophy. The varying dependency on light duration in these two metabolic modes of *O. gloeopara* may be attributed to the following factors: (1) the addition of organic carbon reduces the sensitivity of osmotrophic *O. gloeopara* to light exposure time, and (2) the increase in light exposure may enhance the demand for light energy by increasing investment in the photosynthetic apparatus. For typical obligate autotrophs, under sufficient light intensity conditions, they usually have an optimal light exposure time for photosynthetic efficiency (43). Once this duration is exceeded, autotrophs’ photosynthetic efficiency may decline due to potential photoinhibition (44). Under this circumstance, due to the high plasticity of their metabolic modes, mixotrophs may adjust resource allocation between organic carbon assimilation and photosynthetic processes according to environmental light conditions (45). In our study, osmotrophic *O. gloeopara* showed a shift toward greater reliance on autotrophic metabolism as the light duration increased.

Moreover, the reduced non-photochemical quenching (NPQ) observed in osmotrophic *O. gloeopara* further supported that *O. gloeopara* tended to be more autotrophic under prolonged light exposure (46). When the light exposure duration exceeds 14 h, NPQ of autotrophic *O. gloeopara* increased, demonstrating that under prolonged light conditions, autotrophic *O. gloeopara* reduced light-induced damage by enhancing heat dissipation. For mixotrophs with heterotrophy preference, their photosynthetic efficiency in autotrophic status is usually lower than that of obligate autotrophs in order to reduce high investment on low-income photosynthesis (47), which can help maintain population levels under resource-limited environments (48). Nevertheless, as light exposure time increased, autotrophic *O. gloeopara* not only exhibited reduced photosynthetic efficiency but also decreased chlorophyll content, thereby lowering the metabolic cost of photosynthesis. Consequently, the photosynthetic performance of osmotrophic *O. gloeopara* under prolonged light exposure gradually approached that of autotrophic *O. gloeopara*, indicating a convergence in photosynthetic strategy in response to photoperiod extension.

### Carbon acquisition pathways in *O. gloeopara* under photoperiod variation

The relative contributions of autotrophic and heterotrophic metabolism in mixotrophs are influenced not only by species-specific factors (49, 50) but also by environmental changes (51). In particular, organisms capable of flexible carbon acquisition often adjust their reliance on different metabolic pathways in response to environmental variation. In this study, prolonged light exposure led to a decrease in osmotrophic carbon acquisition by *O. gloeopara*. Consistently, a parallel grazing experiment revealed a significant decline in the grazing rate on algal cells, resulting in a lower contribution of food-derived organic carbon. This shift may be due to the fact that photosynthesis, as the primary energy acquisition pathway for mixotrophic organisms, becomes more efficient with prolonged light exposure, thereby increasing energy levels and reducing dependence on heterotrophic energy and nutrient acquisition. Furthermore, under high-intensity grazing conditions, enhanced mitochondrial metabolism can lead to increased levels of reactive oxygen species (52). While absorbed excess energy during photosynthesis can be dissipated through non-photochemical quenching, the reductive power generated is transferred into mitochondrial metabolism via the malate pathway (23), providing a high-yield energy bypass. Simultaneously, excessive reductive power generated from food decomposition may interfere with critical cellular functions in the mitochondria and induce oxidative damage (53). Therefore, reducing grazing under adequate light conditions is an effective strategy to avoid oxidative stress. With prolonged light exposure, the potential carbon fixation rate of osmotrophic *O. gloeopara* gradually exhibits characteristics similar to those of autotrophic organisms, and the differences between the two conditions become insignificant under full light conditions.

Photoperiod is a stable environmental cue influenced by seasonal and latitudinal variations (54, 55). Many organisms have evolved precise mechanisms for measuring photoperiods to predict and adapt to seasonal transitions (56, 57). For instance, in high-latitude regions, limited light availability during short growing seasons favors efficient photosynthesis and enhanced carbon fixation under extended photoperiods, while in low-latitude regions, organisms adapt to prolonged daylight and higher temperatures (58–60). Our findings suggest that in aquatic ecosystems with prolonged light exposure, primarily autotrophic mixotrophs may typically dominate, as these organisms enhance their photosynthetic capacities to thrive in high-light environments, enabling them to more efficiently fix carbon and gradually transition from being a carbon source to a carbon sink (61). Concurrently, the reduced reliance on external dissolved organic carbon (DOC) further diminishes the competition between mixotrophs and other microbes primarily dependent on DOC for resources. This transition in resource utilization not only enhances the cycling efficiency of inorganic carbon within the ecosystem but also contributes to increased primary productivity (62). Additionally, by reducing the retention of organic carbon within the microbial loop, it fosters the transfer of carbon to higher trophic levels, thereby exerting significant influences on both carbon cycling and energy flow in aquatic ecosystems (63). In natural environments where photoperiod and temperature fluctuate simultaneously, mixotrophs must balance more complex trade-offs between autotrophic and osmotrophic metabolism (64), optimizing energy and nutrient acquisition across seasons. This nutritional flexibility improves their competitive advantage in dynamic environments, allowing them to thrive under varying conditions (45).

The *O. gloeopara* used in this study, isolated from Taihu Lake, a representative natural lake in China, exhibits a marked heterotrophic preference despite possessing functional chloroplasts and retaining photosynthetic capacity. Its carbon acquisition predominantly relies on heterotrophic pathways rather than autotrophy. Previous studies have shown that under environmental stress conditions, enhanced heterotrophic metabolism in *Isochrysis galbana* can effectively compensate for impaired photosynthetic pathways, such as alleviating chronic ultraviolet radiation-induced damage to photosystem II (65). Consequently, the findings of this study are most applicable to mixotrophs with a heterotrophic bias, such as *O. gloeopara*. However, caution is warranted in extending these results to all mixotrophs, as metabolic preferences and strategies exhibit significant diversity among species. Future research could investigate a broader spectrum of mixotrophic organisms under diverse environmental conditions to elucidate the generality and variability of their metabolic strategies and ecological adaptations. Given these metabolic preferences, it is also critical to consider how environmental factors, such as photoperiod and temperature, interact to shape the metabolic responses of mixotrophic organisms. While this study primarily investigates the impact of photoperiod variations on the metabolic response of *O. gloeopara* under controlled temperature conditions to eliminate the confounding effects of temperature fluctuations, in natural environments, temperature and photoperiod changes often occur simultaneously and may interact to influence the metabolic activities of organisms (66). Studies have indicated that temperature elevation typically enhances the heterotrophic activity of mixotrophic organisms. Thus, in natural ecosystems, rising temperatures are often accompanied by longer photoperiods, which may result in lower autotrophic metabolic efficiency compared with the levels observed in our experiments. However, in some specific ecological contexts, photoperiod and temperature changes may not occur simultaneously. For instance, during the spring ice-melting period in high-latitude regions, although light exposure time gradually increases, temperatures remain low (67). Similarly, in some anthropogenically influenced environments, such as lakes or reservoirs, localized temperature increases and artificial light sources at night leading to disrupted natural photoperiods may also induce complex metabolic responses (68, 69). These phenomena highlight the complexity of the interaction between photoperiod and temperature. Therefore, future studies should incorporate temperature as a covariate to further investigate the combined effects of photoperiod and temperature interactions on the metabolic patterns of mixotrophic organisms.

In summary, this study reveals that photosynthetic efficiency in autotrophically grown *O. gloeopara* is modulated by light exposure time, while the addition of organic carbon disrupts this rhythmicity. Under autotrophic conditions, *O. gloeopara* reached optimal photosynthetic efficiency at a light exposure time of approximately 12 h, and as light duration increased, both photosynthetic investment and efficiency decreased, while their thermal dissipation increased to prevent photodamage. As a result, autotrophic *O. gloeopara* tended to develop smaller cell volumes and exhibited more rapid population growth rate with prolonged light exposure time. Under osmotrophic conditions, *O. gloeopara* adapted to prolonged light exposure time by reducing the reliance on external organic carbon sources and increasing investment in the photosynthetic machine as well as decreasing photosynthetic efficiency. Ultimately, they achieved rapid population increase through more efficient energy acquisition via autotrophic metabolism. These findings advance our understanding of the response to photoperiodic changes in osmotrophic *O. gloeopara* and offer valuable insights into their population dynamics and ecological roles in natural environments.

## MATERIALS AND METHODS

### Microorganism cultivation

In this study, *Ochromonas gloeopara* originally isolated from Meiliang Bay (31°24′N, 120°13′E), Taihu Lake, China, was cultivated separately under autotrophic, osmotrophic, and heterotrophic conditions (70). Microalgae *Microcystis aeruginosa* (FACHB-2213) purchased from the Freshwater Algae Culture Collection of the Institute of Hydrobiology, China, was used as an organic carbon resource. Both *O. gloeopara* and *M. aeruginosa* were cultured in axenic BG-11 medium in an incubator at 25°C under 60 µmol m^−2^ s^−1^ photons with a 14 h:10 h light: dark cycle. Prior to the experiment, all the organisms were maintained in the exponential growth phase. Before being used as prey in the experiment, *M. aeruginosa* was centrifuged and re-suspended in sterile BG-11 medium, and then was heat killed at 80°C for 30 min.

### Experimental design

#### Experiment 1

In this experiment, *O. gloeopara* were separately adapted in experimental conditions for 10 days prior to the experiment (Fig. S1). There were seven different photoperiod treatments, including light: dark cycle of 24 h:0 h, 20 h:4 h, 14 h:10 h, 12 h:12 h, 8 h:16 h, 4 h:20 h, and 0 h:24 h at 25°C. Light intensity in this study is 60 µmol m^−2^ s^−1^ photons. Through setting up different availabilities of light and prey, *O. gloeopara* was cultured under different nutritional conditions, including (i) autotrophic growth under only light exposure, (ii) osmotrophic growth under the exposure of both light and sufficient organic matter, and (iii) heterotrophic growth with only sufficient organic matter. Glucose as the organic matter was used in this experiment with the addition of 100 mg L^−1^ and was supplemented every 24 h during the experiment in order to ensure the nutritional status of *O. gloeopara* in osmotrophic and heterotrophic modes. To avoid the influence of high-density populations, *O. gloeopara* grown under heterotrophic and osmotrophic conditions was re-inoculated on the 5th day at corresponding conditions.

After adaptation, *O. gloeopara* grown under different metabolic modes and different photoperiods was respectively cultured at corresponding conditions with the initial concentration of ~2.0 × 10^4^ cells mL^−1^ at 25°C. Under light exposure, light intensity is 60 µmol m^−2^ s^−1^ photons. All the treatments were run in triplicate and performed in 250 mL flasks containing 150 mL of sterilized BG-11 medium. Heterotrophic *O. gloeopara* was cultured in a tinfoil-wrapped flask in a dark incubator, and the samples were kept away from light when sampling. Then, 100 mg L^−1^ of glucose as organic carbon was supplemented to *O. gloeopara* every 24 h in both osmotrophic and heterotrophic modes. The experiment lasted for 10 days. Photosynthetic efficiency, chlorophyll *a* content, and cell volume were tested at the exponential phase (i.e., 48 h). Glucose concentrations and the population abundances of *O. gloeopara* were determined daily. Samples for cell counts were fixed with 2% Lugol’s solution and counted using a 0.1 mm^3^ hemocytometer under a microscope. Each treatment was performed in triplicate, and all the flasks were shaken three times every day.

#### Experiment 2

Considering ingesting organic particles as a main way of carbon acquisition in *Ochromonas*, this study additionally assessed the ingestion rate of *O. gloeopara* under different photoperiods to serve as a supplementary analysis to evaluate the potential contribution of heterotrophic carbon intake. The grazing rates of *O. gloeopara* were calculated using heat-killed *Microcystis* (FACHB-2213) as prey during 48 h. Each flask was inoculated with *O. gloeopara* to a final density of 2 × 10^4^ cells mL^−1^ and heat-killed *Microcystis* to a saturated prey abundance of 3 × 10^6^ cells mL^−1^. The population size of *O. gloeopara* and *Microcystis* was fixed with 2% Lugol’s solution and counted every 12 h using a hemocytometer under a microscope.

To calculate the ingestion rate of *O. gloeopara*, samples for cell counting were obtained after 0, 12, 24, and 48 h and preserved in Lugol’s solution (2%). The ingestion rate (*I,* prey *O. gloeopara*^−1^ d^−1^) was calculated from the difference in prey decay rate in the control (*μ_pc_,* d^−1^) and the grazing treatment (*μ_pt_,* d^−1^), *I* = (*μ_pc_-μ_pt_*)×*N_p_*/*N_o_*, where *Np* and *No* are the average abundances of prey and *O. gloeopara* in grazing treatment, respectively.

### Growth rate and photosynthetic performances

The population dynamics were fitted using the logistic model *N* = *K*/(1+$e^{a-rt}$) to obtain the population growth rate (*r,* d^−1^). Photosynthetic performances of photosystem II and chlorophyll *a* (Chla) contents of *O. gloeopara* during the experiments were measured using Phyto-PAM (Walz, Germany). In this study, the detected photosynthetic performances included *Fv/Fm*, *ΦPSII*, ETRmax, αETR, NPQ, and photosynthetic carbon fixation rate. The photosynthetic carbon fixation rate (*PCFR*, pmol *C* cell^−1^ h^−1^) was calculated following the method of Wilken et al. (29): $PCFR=0.5\times \overset{-}{a^{*}}\times Chla/cell\times L\times \Phi PS_{II}\times 0.25$, where the coefficients “0.5” indicated the coefficient related to two electron excitations (at PSI and PSII) and a maximum electron yield (fixed carbon content) per electron, $\overset{-}{a^{}}$ is the spectrally averaged (400–700 nm) chlorophyll-specific absorption cross section, L was the light intensity, and the coefficients “0.25” indicated the maximum electron yield of (fixed carbon content) per electron.

### Cell volume and morphology

Cell volumes and morphology of *O. gloeopara* were measured at the exponential phase using an image analysis system (Nikon Digital Sight DS-U3; Tokyo, Japan) under microscope (Nikon Eclipse Ci-S; Tokyo, Japan). As *O. gloeopara* is approximately spindle in shape, the cell volume (*V*) was determined by the cell length (*L*) and width (*W*): *V* = *π* (*W*^2^ ×*L*)/6.

### Glucose concentration

Glucose concentration was determined by the glucose oxidase-peroxidase method using a commercially available glucose assay kit (Jiancheng F006, Nanjing, China). The organic carbon utilization rate (OCR, pmol C per *Ochromonas* per hour) was determined by measuring the glucose consumption by the average abundances of *O. gloeopara* at the exponential phase over 24 h. All treatments in this study were performed under sterile conditions to prevent bacterial interference.

### Statistical analysis

All data were presented as mean ± SE. The maximum abundance, photosynthetic performances, and cell volume were fitted using the Gaussian distribution model $Y=ae^{\left(-0.5{\left(\frac{x-x_{0}}{b}\right)}^{2}\right)}$ to obtain the maximum value a, and the light exposure time of the maximum value $x_{0}$. The effect of photoperiod on the dynamic changes of photosynthetic efficiency and population dynamics of *O. gloeopara* was analyzed by repeated measurement variance analysis. Two-way ANOVA was used to test the effects of the photoperiods and nutritional modes on *O. gloeopara* performances. Tukey’s post-hoc comparison was performed, while significant differences were detected among treatments. We used R statistical software (version 1.1.463 with *R* Studio) for Spearman correlation analysis (Package *ggcorrplot*). The other analyses were performed by SigmaPlot 14.0. The effect size (Hedges’ g ±95% CI) was used to assess the impact of different photoperiods on photosynthetic efficiency of *O. gloeopara* under different nutritional modes following the method of Hedges and Olkin (71).

## SUPPLEMENTAL MATERIAL

Supplemental material aem.01032-25-s0001.docx Figure S1 and Table S1.

ASM does not own the copyrights to Supplemental Material that may be linked to, or accessed through, an article. The authors have granted ASM a non-exclusive, world-wide license to publish the Supplemental Material files. Please contact the corresponding author directly for reuse.
