# Supplementary material for: Prolonged light exposure time enhances the photosynthetic investment in osmotrophic Ochromonas
Source: Appl Environ Microbiol. 2025 Jun 24;91(7):e01032-25. doi: 10.1128/aem.01032-25 (PMC12285262; doi:10.1128/aem.01032-25)
Supplement: Supplemental material — Figure S1 and Table S1. [file aem.01032-25-s0001.docx]

**Supporting Information**

**Prolonged light exposure time enhances the photosynthetic investment in osmotrophic *Ochromonas***

Xiaoqing Xu, Xiaoyu Cheng, Zhihao Shao, Zhou Yang, Lu Zhang*

Jiangsu Key Laboratory for Biodiversity and Biotechnology, School of Biological Sciences, Nanjing Normal University, 1 Wenyuan Road, Nanjing 210023, China

*Address correspondence to Lu Zhang, [zhanglu@njnu.edu.cn](mailto:zhanglu@njnu.edu.cn)

**Other authors’ email addresses:**

Xiaoqing Xu, e-mail: 9096[6@njnu.edu.cn](mailto:6@njnu.edu.cn)

Xiaoyu Cheng, e-mail: 231202150@njnu.edu.cn

Zhihao Shao, e-mail: [231202139@njnu.edu.cn](mailto:231202139@njnu.edu.cn)

Zhou Yang, e-mail: [yangzhou@njnu.edu.cn](mailto:yangzhou@njnu.edu.cn)

Number of pages: 4

Number of figures: 1

Number of tables: 1

**Supplemental Figures**

**FIG S1. Diagram of the experimental design.**

**Supplemental Tables**

**Table S1.** Two-way ANOVA of the effects of photoperiod and nutritional mode on relevant indices of *Ochromonas* (Gray background: Growth-related indices; Blue background: Photosynthetic efficiency-related indices; Green background: Carbon fixation-related indices).

| **Effects** | **Responses** | **DF** | **SS** | **MS** | **F** | ***P*** |
| --- | --- | --- | --- | --- | --- | --- |
| Nutritional modes (a) | Population growth rate | 1 | 3.631 | 3.631 | 198.163 | <0.001 |
| Light exposure time (b) |  | 4 | 0.0213 | 0.00533 | 0.291 | 0.881 |
| a x b |  | 4 | 0.507 | 0.127 | 6.922 | 0.001 |
| Nutritional modes (a) | Maximum abundance | 1 | 576343.153 | 576343.153 | 2986.406 | <0.001 |
| Light exposure time (b) |  | 4 | 72421.216 | 18105.304 | 93.815 | <0.001 |
| a x b |  | 4 | 44667.29 | 11166.822 | 57.863 | <0.001 |
| Nutritional modes (a) | Time to reach maximum density | 1 | 0.3 | 0.3 | 1.8 | 0.195 |
| Light exposure time (b) |  | 4 | 19.133 | 4.783 | 28.7 | <0.001 |
| a x b |  | 4 | 13.533 | 3.383 | 20.3 | <0.001 |
| Nutritional modes (a) | Cell volume | 1 | 436492.937 | 436492.937 | 106.47 | <0.001 |
| Light exposure time (b) |  | 4 | 530533.495 | 132633.374 | 32.352 | <0.001 |
| a x b |  | 4 | 454006.209 | 113501.552 | 27.685 | <0.001 |
| Nutritional modes (a) | *Chl a* | 1 | 0.473 | 0.473 | 194.71 | <0.001 |
| Light exposure time (b) |  | 4 | 0.129 | 0.0323 | 13.315 | <0.001 |
| a x b |  | 4 | 0.223 | 0.0558 | 22.983 | <0.001 |
| Nutritional modes (a) | Fv/Fm | 1 | 0.0975 | 0.0975 | 50.503 | <0.001 |
| Light exposure time (b) |  | 4 | 0.0916 | 0.0229 | 11.859 | <0.001 |
| a x b |  | 4 | 0.09 | 0.0225 | 11.664 | <0.001 |
| Nutritional modes (a) | ΦPSII | 1 | 0.0563 | 0.0563 | 38.497 | <0.001 |
| Light exposure time (b) |  | 4 | 0.146 | 0.0365 | 24.946 | <0.001 |
| a x b |  | 4 | 0.0309 | 0.00772 | 5.279 | 0.005 |
| Nutritional modes (a) | aETR | 1 | 0.0108 | 0.0108 | 63.718 | <0.001 |
| Light exposure time (b) |  | 4 | 0.0189 | 0.00473 | 27.802 | <0.001 |
| a x b |  | 4 | 0.00438 | 0.0011 | 6.447 | 0.002 |
| Nutritional modes (a) | NPQ | 1 | 0.323 | 0.323 | 24.454 | <0.001 |
| Light exposure time (b) |  | 4 | 0.313 | 0.0783 | 5.929 | 0.003 |
| a x b |  | 4 | 0.31 | 0.0774 | 5.858 | 0.003 |
| Nutritional modes (a) | Carbon fixation rate | 1 | 0.00545 | 0.00545 | 18.043 | <0.001 |
| Light exposure time (b) |  | 4 | 0.022 | 0.00549 | 18.197 | <0.001 |
| a x b |  | 4 | 0.00688 | 0.00172 | 5.696 | 0.003 |
| Nutritional modes (a) | Ingestion rate | 1 | 0.3 | 0.3 | 1.8 | 0.195 |
| Light exposure time (b) |  | 4 | 19.133 | 4.783 | 28.7 | <0.001 |
| a x b |  | 4 | 13.533 | 3.383 | 20.3 | <0.001 |
